# Persistent Buccal Swelling: Diagnostic Dilemma

**DOI:** 10.7759/cureus.3722

**Published:** 2018-12-11

**Authors:** Malarkodi Thanthoni, Sathasivasubramanian S, Aravind Warrier S

**Affiliations:** 1 Oral Medicine and Radiology, Faculty of Dental Sciences, Sri Ramachandra University, Chennai, IND; 2 Oral Medicine & Radiology, Faculty of Dental Sciences, Sri Ramachandra University, Chennai, IND

**Keywords:** buccal swelling, lipoma, dental abscess

## Abstract

Taking a systematic and comprehensive patient history is undoubtedly an essential tool for a proper diagnosis. We report a unique case of a patient presenting with persistent buccal swelling, a rare presentation of two unrelated pathologies occurring concurrently. In this case, the presence of a lipoma was concealed by a dental abscess and an incomplete patient history.

## Introduction

Lipomas are benign tumors composed of a well-circumscribed mass of mature adipose tissue [1]. Although it is one of the more common mesenchymal neoplasms to occur elsewhere in the body (such as the trunk, shoulder axilla, etc.), a lipoma is relatively uncommon in the submandibular region [2]. Lipomas occurring in the head region account for 15% to 20% of cases [3], and the peak incidence is during a patient’s fifth or sixth decade of life [4]. Lipomas usually present as asymptomatic, slow-growing masses, and the clinical course can last for several years [5]. We report an uncommon presentation of a lipoma occurring in the submandibular region in a 53-year-old South Indian man.

## Case presentation

**Figure 1:**
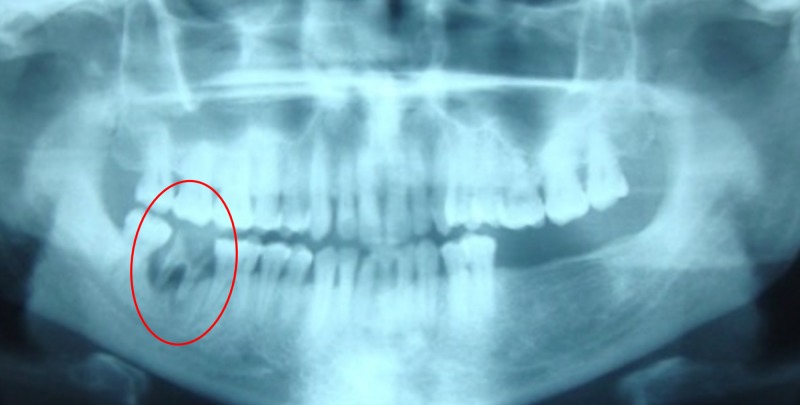
Orthopantomogram shows radiolucency in tooth 47 with gross destruction of the crown associated with radiolucency around the roots

A 53-year-old man sought treatment at the department of oral medicine and radiology at Sri Ramachandra University in Chennai, India, for a painful and progressive swelling involving the right mandibular region for four days. It was associated with a toothache on the right lower jaw. The pain in the tooth was dull, continuous, and aggravated on taking hot or cold beverages. The swelling was small when he initially noticed it and gradually increased to the presenting size. The patient had visited a private dentist a day before, where an orthopantomogram was taken, and he was advised to undergo extraction after a course of antibiotics and non-steroidal anti-inflammatory drugs. His medical history was noncontributory. Extra orally, a single, well-defined swelling was evident in the right lower jaw region, with signs of inflammation. The swelling was 4 cm x 5 cm, warm, tender, soft, compressible, and not fluctuant. Intraoral examination revealed dental caries in 47, with gross destruction of the crown and mucobuccal fold obliteration in relation to 47. A provisional diagnosis of dental caries in 47, with a dentoalveolar abscess, was promptly made. The previous orthopantomogram revealed radiolucency in 47 with gross destruction of the crown associated with radiolucency around the roots (Figure 1). No other associated pathologies were evident. The patient was advised to continue the same medication for four more days. Four days later, the patient reported with painless swelling, which had reduced in size to 3 cm x 4 cm involving the same site. The swelling was nontender, soft, and compressible; it was not fluctuant, pulsatile, or fixed to the skin and underlying bone. The results of the transillumination screening were negative.

**Figure 2:**
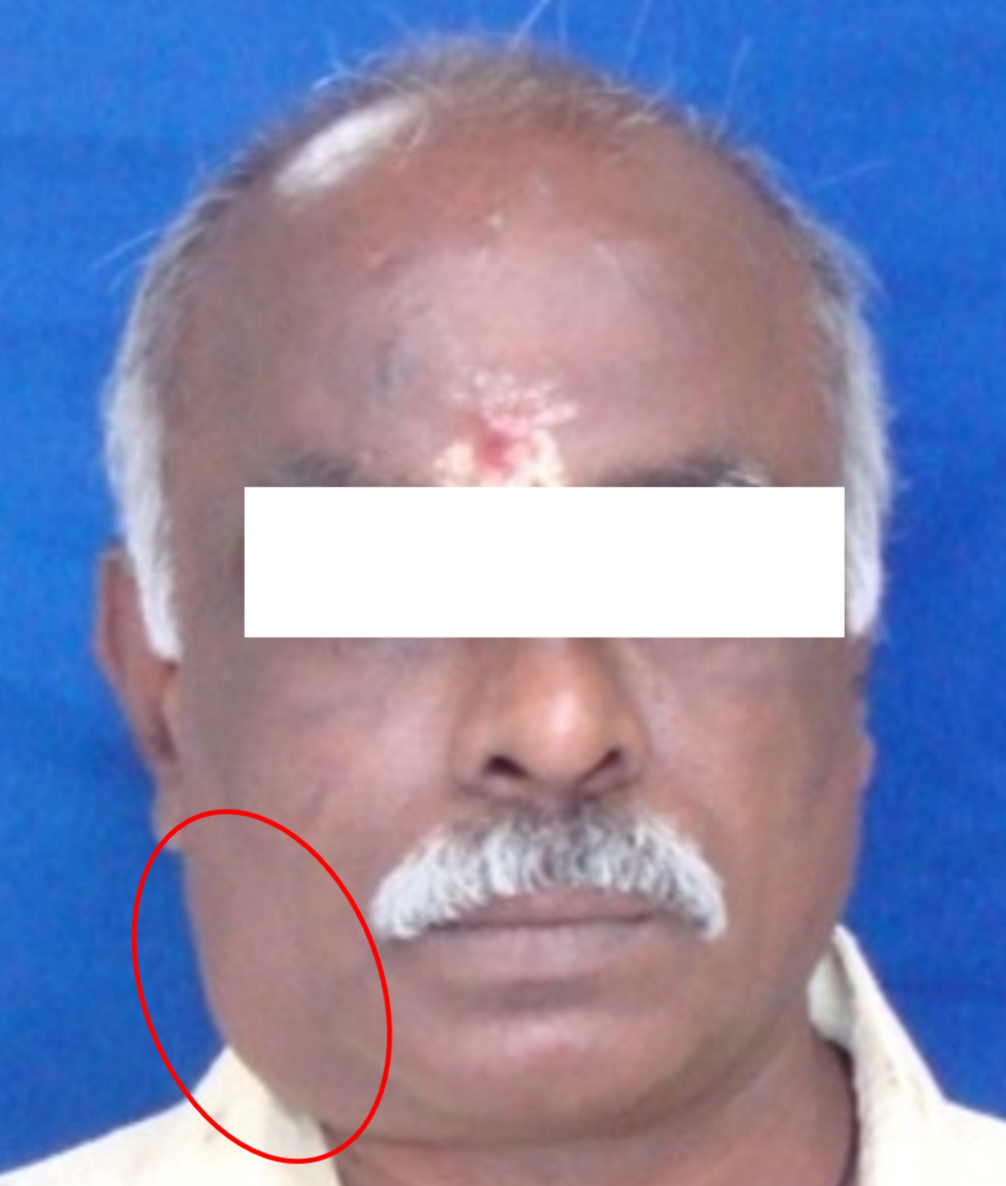
Persistent buccal swelling in the right mandibular region after the extraction of tooth 47

Given that the patient was asymptomatic, we extracted tooth 47 while he was under local anesthesia. The postoperative period was uneventful. When the patient was evaluated one week later, the asymptomatic swelling remained the same size (Figure 2).

**Figure 3:**
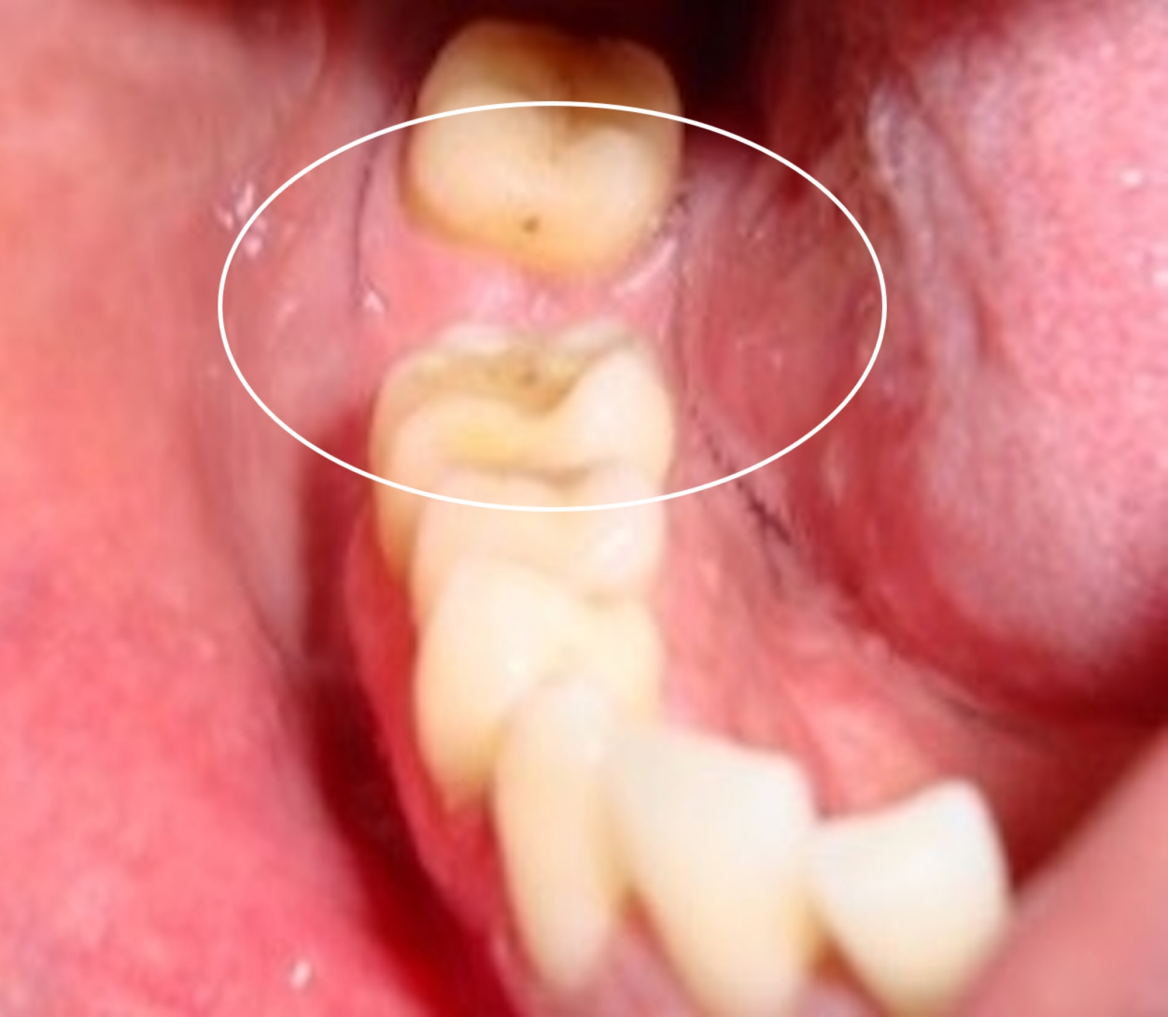
Intraoral picture after the extraction of tooth 47 shows normal mucosa

There was no cervical lymphadenopathy and, intraorally, the mucosa was normal and moist (Figure 3). The mouth opening was 4.2 cm inter incisally. The patient could vaguely recollect the presence of a painless small lump for one year, which was brought to his notice by his family members. The patient did not experience pain at any time, nor was there any change in the size of the swelling before and after eating.

**Figure 4:**
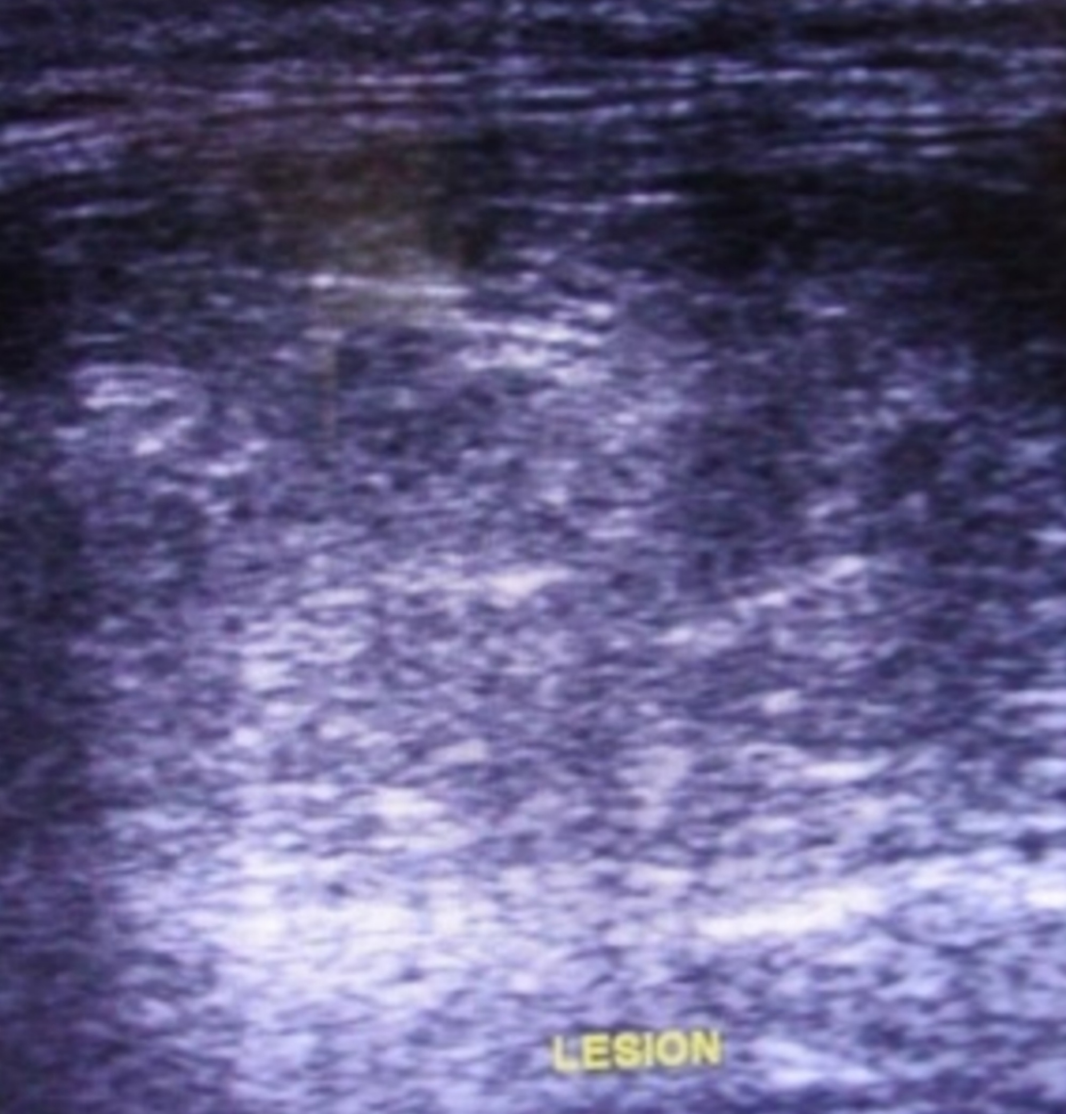
Ultrasonography shows a well-circumscribed hyperechoic mass

Considering the history of painless swelling lasting one year that was well-defined, soft, nontender, with no intraoral manifestation, a lipoma of the right submandibular region was suspected. Fine needle aspiration cytology was performed under ultrasound guidance. Ultrasonography revealed a well-circumscribed hyperechoic mass measuring 3.2 cm x 2.7 cm separated from the right submandibular gland (Figure 4).

**Figure 5:**
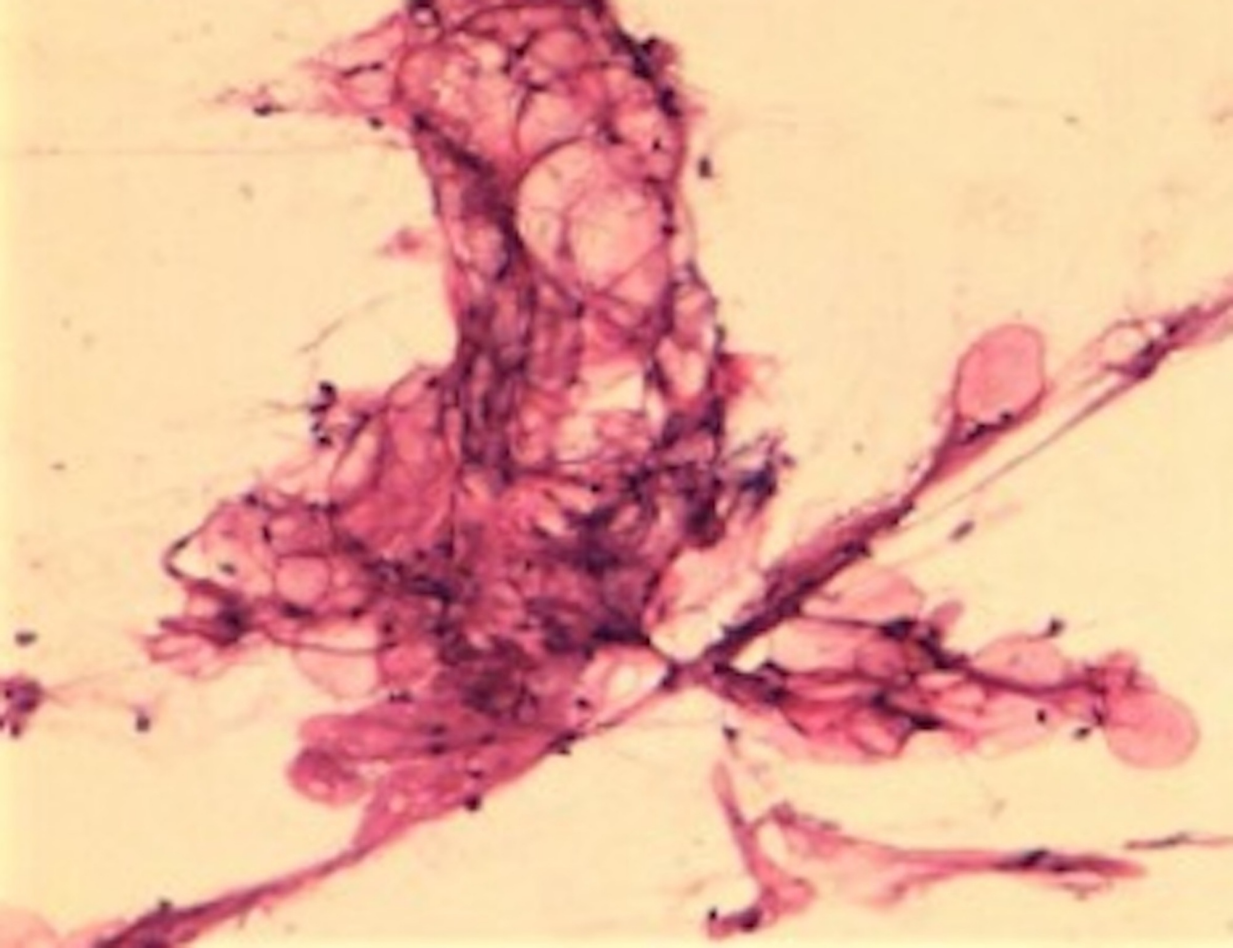
Aspirates show mature fibro-adipose tissue

The smear showed mature fibro-adipose tissue, which would be consistent with a lipoma (Figure 5).

**Figure 6:**
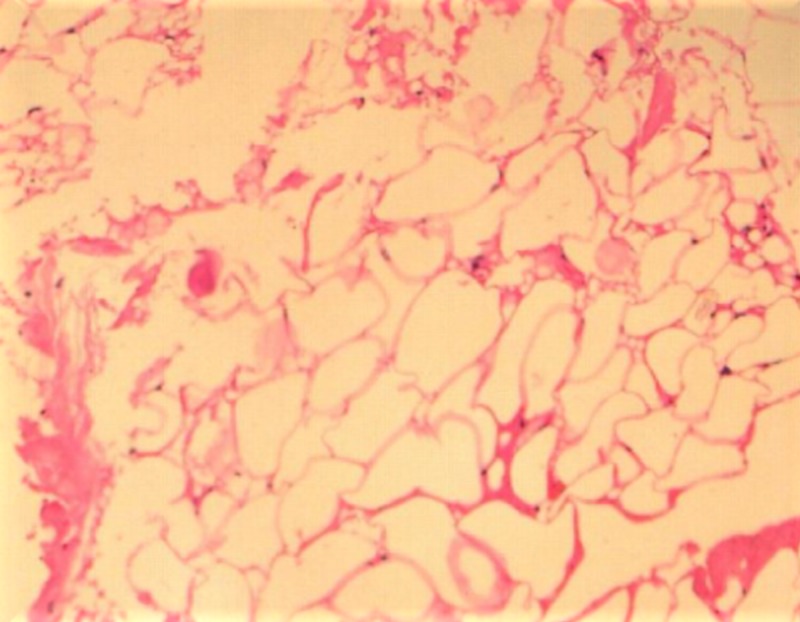
Photomicrograph showing mature adipocytes arranged in a lobular pattern with a few capillaries and fibro-collagenous tissue

Surgical removal of the lesion was done by the submandibular gland approach under general anesthesia. The excised mass showed mature adipocytes, a few capillaries, and fibro-collagenous tissue. The physical features were again consistent with a lipoma (Figure 6).

## Discussion

A complete and accurate history is the basis for an effective care plan. Despite today’s medical advancements, a patient’s history remains the mainstay for diagnosis. This case presented a rare situation where two distinct, unrelated pathologies (a dental abscess and a lipoma) coexisted, invariably making the clinical course complex. While the buccal aspect of the mandibular body remains an infrequent site for lipomas, its possible presence was masked by the unambiguous signs of odontogenic infection and an incomplete patient history, which naturally led to considerable uncertainty about the source of persistent swelling.

Lipomas are benign tumors of soft tissue origin occurring from mature adipose tissue [1]. It is the most common mesenchymal neoplasm and constitutes 5% of all benign tumors of the body [6]. The shoulder, back, arm, anterior chest wall, and axilla are the most common sites [2]. Presentation in the head and neck region is relatively rare and constitutes approximately 15% to 20% cases. The posterior neck and parotid region are more frequently involved sites [3]. The peak incidence is between the fifth and sixth decade of life [4]. Clinically, lipomas present as soft, painless, slow-growing, well-delineated mobile masses [5].

According to World Health Organization, benign adipocytic tumors are classified into “Lipoma, Lipomatosis, Lipomatosis of nerve, Lipoblastoma/lipoblastomatosis, Angiolipoma, Myolipoma of soft tissue, Chondroid lipoma, Extra-renal angiomyolipoma, Extra-adrenal myelolipoma, Spindle cell/pleomorphic lipoma, and Hibernoma” [7]. The cause of a lipoma remains uncertain. Various pathogenic mechanisms like origin from lipoblastic embryonic cell nest, metaplasia of muscle cells, fatty degeneration, trauma, and infection have been proposed as plausible causative factors [8-10].

Given that a lipoma is a soft tissue neoplasm, ultrasonography (USG) and magnetic resonance imaging (MRI) serve as valuable diagnostic tools. Although lipomas show variable echogenicity, they are usually hyperechoic, containing linear echogenic lines under USG [11]. In T1-weighted MRI images, a lipoma appears as a high-intense mass and with moderate intensity in T2-weighted images [1].

Histologically, a lipoma is composed of mature adipocytes arranged in a lobular pattern usually surrounded by a thin fibrous capsule. Although they resemble normal fat cells, they are distinguished from normal adipocytes by the fact that they are not utilized for metabolism. Surgical excision remains the mainstay of treatment, and the recurrence rate is very low [2,12].

## Conclusions

Our patient presented with dental pain and an abscess in the right lower jaw region, a site with a preexisting lipoma. The presence of the lipoma was concealed by the definite signs of a dental abscess. Though the features of lipomas are usually straightforward, systematic clinical examination and diagnostic workup are essential for successful management. We were fortuitous to handle both the abscess and the lipoma successfully, without causing discomfort to the patient. However, we may not always be so fortunate. Therefore, accurate and detailed information regarding a patient’s history is indispensable for optimal patient care.
